# Patient and clinician views on inpatient antibiotic shared decision-making: a qualitative study

**DOI:** 10.1093/jacamr/dlaf228

**Published:** 2026-01-07

**Authors:** C Roleston, M Wanat, F Mowbray, J Underhill, M Wilcock, S Jacklin, J Amos, K B Bamford, S Hughes, N Marsh, S Tonkin-Crine, N Powell

**Affiliations:** Nuffield Department of Primary Care Health Sciences, University of Oxford, Oxford, UK; Nuffield Department of Primary Care Health Sciences, University of Oxford, Oxford, UK; National Institute for Health Research (NIHR) Health Protection Research Unit (HPRU) in Healthcare Associated Infections and Antimicrobial Resistance, University of Oxford, Oxford, UK; Nuffield Department of Primary Care Health Sciences, University of Oxford, Oxford, UK; School of Allied Health Professions and Pharmacy, Keele University, Keele, Staffordshire, UK; Pharmacy Department, Royal Cornwall Hospital Trust, Truro, Cornwall, UK; School of Allied Health Professions and Pharmacy, Keele University, Keele, Staffordshire, UK; Pfizer, Tadworth, Surrey, UK; Clinical Microbiology, Royal Cornwall Hospital Trust, Truro, Cornwall, UK; Pharmacy Department, Chelsea & Westminster NHS Foundation Trust, London, UK; Pharmacy Department, Royal United Hospitals Bath NHS Foundation Trust, Bath, UK; Nuffield Department of Primary Care Health Sciences, University of Oxford, Oxford, UK; National Institute for Health Research (NIHR) Health Protection Research Unit (HPRU) in Healthcare Associated Infections and Antimicrobial Resistance, University of Oxford, Oxford, UK; Pharmacy Department, Royal Cornwall Hospital Trust, Truro, Cornwall, UK

## Abstract

**Background:**

Shared decision making (SDM) is a collaborative process between patients and prescribers and identified as a strategy to support antimicrobial stewardship. SDM can improve patient and clinician satisfaction and reduce antibiotic prescribing. However, little is known about how to implement antibiotic SDM in secondary care.

**Objectives:**

Identify opportunities for antibiotic SDM between patients and clinicians in secondary care.

**Methods:**

Semi-structured interviews were conducted with senior decision makers (registrar or consultant grade) and adult patients who had received antibiotics during their medical or surgical admission, recruited from three secondary care Trusts in England. Interviews explored participants’ views on opportunities for SDM when prescribing antibiotics in secondary care, guided by the ‘Start Smart, Then Focus’ framework. Interviews were audio recorded, transcribed verbatim and analysed thematically.

**Results:**

18 clinicians and 20 patients were interviewed. Two themes were identified. In ‘Pushing back against SDM’, participants challenged the amenability and prioritization of SDM for antibiotics in inpatient settings, related to clinicians being seen as main decision makers, with patients not seeking further involvement. This was reinforced by the perceived urgency of treatment, the fast-paced hospital environment, and the view that antibiotic decisions were either too complex or too straightforward to invite shared input. In ‘If not SDM, then what?’, participants endorsed bi-directional communication and information provision as alternative priorities, highlighting its value.

**Conclusions:**

SDM was not well understood or endorsed for antibiotic prescribing decision making in secondary care. Further work is warranted to educate and upskill clinicians in SDM as a concept within secondary care.

## Introduction

Antimicrobial resistance (AMR) is a leading cause of death globally^1^ and is increasingly recognized as a public health emergency.^2^ Inappropriate antibiotic use is a key driver of AMR.^3,4^ ‘Patient engagement’ has been identified as a potential strategy to supplement antimicrobial stewardship (AMS) efforts^5^ with shared decision making (SDM) an opportunity to expand the role of patients in AMS initiatives.

SDM aims to facilitate collaboration between patients and prescribers to work together and reach a joint decision about treatments on the basis of available evidence and patient preferences, beliefs, circumstances and values.^6^ SDM is an ambition of the UK Department of Health, and NICE have produced SDM guidance to promote implementation in everyday care in all healthcare settings.^6^

While there is recognition that receptiveness to SDM may differ between patients, it has been argued there is an ethical imperative for patients to be more involved in care decisions.^7^ Accumulating evidence links SDM with benefits including self-reported patient knowledge, patient satisfaction and trust in healthcare professionals^8,9^ and clinician satisfaction without increasing consultation time.^10^ A Cochrane review^11^ concluded there is moderate quality evidence SDM reduces antibiotic prescribing. SDM within the context of secondary care has largely focused on breast cancer treatment and surgery decisions^8^ with scant attention afforded to antibiotic prescribing in this setting.

‘Start Smart, Then Focus’ (SSTF) is an AMS framework developed by the UK Department of Health.^12^ SSTF provides evidence-based guidance for secondary care clinicians to reduce the risk of AMR while safeguarding patient care. The SSTF framework does not explicitly include patient perspective and so is naturally a clinician-led tool. In practice, ‘start smart’ involves establishing clear evidence of infection, initiating appropriate microbiological investigations, initiating prompt antimicrobial prescribing compliant with local guidelines and with sufficient documentation. It then involves review and revision of antibiotic prescriptions within 48–72 hours around five key actions: (i) stopping antibiotics; (ii) switching from IV to oral; (iii) changing antibiotics; (iv) continuing antibiotic and (v) move to outpatient parenteral antimicrobial therapy.

Despite appetite for SDM^13^ related to AMS efforts in hospital settings,^14^ the unique demands of hospital environments^15^ warrant further exploration of SDM in secondary care. We aimed to identify opportunities for antibiotic prescribing SDM between patients and clinicians in secondary care using the SSTF framework to scaffold discussion.

## Methods

### Design

Qualitative study using semi-structured interviews with patients and clinicians in secondary care Trusts in England.

### Recruitment

Three secondary care Trusts in England were purposefully identified to capture rural (Site A), urban/rural (Site B) and urban (Site C) geographies that serve different patient populations (e.g. ethnicity, age, deprivation). All prospective participants were identified and recruited from within these sites. Two groups were recruited.

Senior decision makers (registrar or consultant grade) working in medical or surgical adult specialities in participating NHS hospital trusts were eligible for participation. Email invitations with the Participant Information Sheet attached were sent to medical and surgical leads to cascade within their teams who were asked to contact the Principal Investigator if they were interested in participating.

Adult patients who had received antibiotics for an acute infection during their current medical or surgical admission were eligible. Pharmacy staff either identified eligible patients when dispensing discharge medication and placed a Participant Information Sheet in with their discharge medication, or identified eligible patients on ward rounds, verbally explained the nature of the study and provided a Participant Information Sheet. Patients were asked to contact the Principal Investigator if they would like to participate in the study. Qualitative researcher(s) then contacted patients (telephone/email) to recruit them to the study.

We provisionally sought to complete 50 interviews across the two groups from May 2024 to March 2025. This was reviewed iteratively throughout data collection informed by the concept of informational power where data collection ceased when data of sufficient quality had been generated to answer the research questions.^16^ Patients received £20 of shopping vouchers in line with NIHR guidance, while clinicians received £40 of shopping vouchers in line with the standard hourly rate.

### Data collection

Semi-structured interviews were chosen to gain in-depth insights about the chosen phenomenon.^17^ In line with the approach, a topic guide for each group was developed on the basis of the primary research question and informed by the SSTF framework^12^ [see the Supplemental File (available as Supplementary data at *JAC-AMR* Online)]. Interviews with clinicians and patients explored their views on whether there is opportunity for SDM when prescribing antibiotics in secondary care using the SSTF framework as a guide.

Verbal consent was obtained from participants before interviews were conducted. Interviews were conducted online (Microsoft Teams) or over the telephone by experienced qualitative researchers (PhD qualified with substantial previous experience conducting qualitative health research), were audio recorded and transcribed verbatim by an independent transcription company. Researchers and participants were unknown to one another.

### Data analysis

Data collection and analysis were performed concurrently. Data from all interviews were analysed using an inductive thematic analysis.^18^ Initial coding was performed separately on patient (by C.R.) and clinician (by M.W. and F.M.) data. The first four transcripts for each group were used to develop a coding framework, which was then applied to the remaining transcripts in that group. Changes were made to the framework iteratively. The preliminary coding frameworks operated at the categorical level that were then discussed to identify points of convergence and divergence across and within participant groups. This discussion precipitated the development of conceptual themes that captured the dialogue between patient and clinician perspectives, which were then further refined in response to additional discussion with the wider team. Finally, for theme two, we used the SSTF framework as an organizational structure to present participants’ priorities.

### Ethics

This study received a favourable decision by the West Midlands—Black Country Research Ethics Committee (REC 23/WM/0059).

## Results

In total, 18 clinicians completed an interview (Site A = 7, Site B = 7, Site C = 4) that lasted 21–52 minutes (mean = 32 minutes). Time in their current role ranged from 2 to 23 years (mean = 7.1 years). In total, 20 patients completed an interview (Site A = 13, Site B = 6, Site C = 1) that lasted 18–90 minutes (mean= 39 minutes). Patients’ age ranged from 30 to 79 years (mean = 54 years), and their hospital stay ranged from 3 to 23 days (mean = 6.9 days). See Tables 1 and 2 for clinician and patient demographics, respectively.

**Table 1.** Clinician demographics

Demographic	Frequency
Site	
A	7
B	7
C	4
Gender	
Male	10
Female	8
Years in role = mean (range)	
7.1 (2–23)	
Role	
Registrar	9
Consultants	9
Specialty	
Medicine	12
Surgical	6

**Table 2.** Patient demographics

Demographic	Frequency
Site	
A	13
B	6
C	1
Gender	
Male	9
Female	11
Age (years)	
30–39	2
40–49	4
50–59	2
60–69	9
70–79	3
Number of hospitalizations in past 2 years	
1	9
2	5
5	2
6	2
7	1
10	1
Length of last hospitalization	
<1 week	9
1 week	6
1–2 weeks	2
2–3 weeks	2
4 weeks+	1

We identified two themes. The first, ‘Pushing back against SDM’, sets out participants’ views challenging the amenability and prioritization of SDM for antibiotics in inpatient settings. In the second, ‘If not SDM, then what?’, participants countered the prioritization of SDM by positing attention should instead be directed towards improving communication and information provision to enhance patient experience and care. Note that no differences were identified between participants based on site, demographics or job role (clinicians).

### Theme 1: pushing back against SDM

#### Sub-theme 1: ownership of power and capability to make decisions

Clinicians viewed themselves as the primary decision makers regarding antibiotic use and typically framed SDM as collaboration between clinicians within a multidisciplinary team. The involvement of patients was limited to checking for allergies, informing patients of the clinician-identified treatment plan, and seeking their consent to initiate it, ‘*it’s [SDM with patients] not something that’s a routine part of my practice to—I often ask patients what they think about discharge or whether they're happy to embrace a certain treatment, or not*’ [Clinician9_SiteB_Surgical Consultant].

Patients likewise characterized decision making to be the purview of clinicians and framed communication with clinicians as directive rather than discursive, ‘*they just told me what I was taking. They didn't ask me*’ [Patient17_SiteA_Female_60 years old]. Or else clinicians administered antibiotic treatment with no communication, ‘*there was really no discussion* (…) *they just put you on it*’ [Patient6_SiteA_Female_62 years old]. Most patients were admitted to Emergency or Intensive Care departments and therefore understood why decisions were made for them initially. However, all patients were in hospital for several days, some weeks, and most recalled ward rounds taking place, typically characterized by unidirectional communication from healthcare professionals despite being well enough to converse and contribute.

Some clinicians believed their patients deferred to their expertise and may resist attempts to include them in decision making, ‘*I think they will just ask why they need to be involved and then they might say, “Well, you're the doctor, you know what bug is treated by what antibiotic. I'll let you decide that.”*’ [Clinician15_SiteA_Surgical Registrar]. Most patients reported having the opportunity to ask questions about their care/treatment with a clinician, but patients seldom asked questions. Reasons for this included, having limited capacity (particularly at admission), not having any questions to ask, perception that clinician did not have time, or did not welcome questions, ‘*you’re not really encouraged to ask questions which I think is pretty normal in my experience doctor-patient relationship because doctor’s know everything, and the public know nothing*’ [Patient2_ SiteA_Male_75 years old]. In addition, patients reported feeling they did not have sufficient knowledge to contribute to decision making and high levels of trust in clinicians’ expertise to decide the best treatment(s) for them. Consequently, most patients deferred to clinicians and did not seek to engage in SDM. However, a minority of patients would have valued a more active role in decision making but felt powerless within the patient–clinician dynamic to influence decision making, ‘*doctors need to remember how powerless you feel in hospital. It can be quite difficult to start a conversation if you do have any worries*’ [Patient8_SiteA_Female_49 years old].

A minority of clinicians saw some merit to SDM that included patient input, ‘*I think adherence to treatment is the main one* [benefit to SDM]’ [Clinician11_SiteC_Medicine Registrar]. However, they still noted the limits to a SDM approach, for example concern patients might ‘*make decisions that we wouldn’t always think were in their best interests*’ [Clinician11] and the challenges communicating complex medical terminology related to antibiotics to enable patients to contribute to SDM.

None of the clinicians in this study had received any formal training in SDM but emphasized ‘*SDM goes hand in hand with good communication and good clinical practice*’ [Clinician7_SiteB_Medicine Consultant], which they argued was routine practice already. Most welcomed SDM training in the future, with only a minority expressing a dissenting view.

#### Sub-theme 2: hospital environment not amenable to SDM

Clinicians and patients frequently identified the patient being too severely unwell and/or lacking capacity to engage in conversation combined with the necessity to initiate treatment(s) quickly limited opportunities for SDM in hospital environments, ‘*the obvious ones would be if the patient lacks capacity and then they’re not going to be able to share their views, and also in scenarios where it’s life or limb saving, I think I would probably recommend giving antibiotics first, then explaining why later if appropriate*’ [Clinician15_SiteA_Surgical Registrar].

A small number of patients felt ‘*intimidated*’ by clinicians within the hospital setting which was not mirrored in their dynamic with clinicians in primary care where they felt more ‘*relaxed*’. Consequently, patients reported higher levels of passive acceptability of treatments while in hospital, ‘*things are actually just happening to you*’ [Patient4_SiteC_Female_68 years old], which they were infrequently motivated or able to challenge.

#### Sub-theme 3: antibiotics not amenable to SDM

Overall, clinicians emphasized the nature of decisions around antibiotics as not being amenable to SDM. They felt their treatment decisions were rooted in robust guidelines and reflected the consensus of colleagues with appropriate expertise, which gave them confidence. Such that, decisions around antibiotics had a clear course of action, unlike other clinical decisions, ‘*we’re going to give the antibiotic that is most appropriate to the organism*’ [Clinician15_SiteA_Surgical Registrar]. Consequently, they believed antibiotic decision making did not require a discussion with patients, and in their view, should be upheld irrespective of patient preferences.

Conversely, clinicians perceived some terminology as too complex: ‘*the terms used to describe organisms, and the antibiotic mechanisms of action are quite complicated and require sort of an underlying basis of knowledge*’ [Clinican15_SiteA_Surgical Registrar] and decisions too nuanced to sufficiently explain to patients: ‘*maybe there is less of an understanding of uncertainty and I think that can be quite a nuanced thing to understand as a patient* (…) *and so maybe there's some difficulty there in kind of how to communicate that*’ [Clinician12_SiteB_Surgical Consultant]. Clinical scenarios that involved seeking advice from microbiology were emphasized as particularly difficult to explain, particularly when clinicians lacked confidence in their own understanding.

Patients likewise viewed the choices for/between antibiotic treatments to be limited, negating the need for them to have any input into the decision, ‘*in terms of antibiotics I can’t see where there would be a shared decision making, a reason to share the decision*’ [Patient2_SiteA_Male_75 years old]. The only exception patients identified related to known side effects that may meaningfully affect them, ‘*I would have wanted to know, well what’s the difference between the two antibiotics. If one says, you get a hairy face and the other one said you get a hairy bum, then I’ll have the hairy bum one, you know* [laughter]’ [Patient13_SiteA_Male_64 years old].

### Theme 2: if not SDM, then what?

As evidenced in the preceding theme, neither clinicians nor patients in this study endorsed SDM in this context. However, both identified bi-lateral communication and information provision to be valuable avenues for more attention to improve patient experience. We have used the SSTF framework as an organizational structure to present the findings for this theme.

#### Admission/initial prescribing

While clinicians acknowledged initial prescribing provided a window of opportunity to start conversations with patients about antibiotics, most emphasized how challenging this would be to implement in practice given the need for rapid decision making. Instead, some clinicians proposed being moved to a ward marked the first opportunity to begin conversations with patients about treatment.

By contrast, admission was most frequently endorsed by patients as the most important time point for information provision. Patients frequently described being in hospital as ‘*disorientating*’ and feeling ‘*out of control*’ and so framed information provision as a mechanism to provide ‘*reassurance*’, ‘*set expectations*’, and respond to patient concerns and beliefs about treatment(s). The latter was particularly salient among patients with more complex or chronic health conditions who were worried about the impact of drug interactions and AMR on their health. While acutely aware of the time pressures faced in hospital settings, patients nevertheless argued investing a small amount of time upfront to educate and reassure patients would establish a solid grounding allowing ‘*it all to go a lot more smoothly*’ [Patient18_SiteB_Female_41 years old].

#### Switching from IV to oral administration

Some clinicians felt that since the switch from IV to oral administration is within the same antibiotic class discussion was not required. In contrast, others felt this was a good opportunity to engage with patients as their condition probably would be sufficiently improved to permit discussion about their condition and treatment, ‘*you’re just explaining a rationale for wanting to switch, you know, infection markers falling, you’ve not spiked a fever for 24, 48 hours, so it should be relatively easy to involve them in the discussion*’ [Clinician13_SiteB_Medicine Registrar].

Overall, patients did not feel strongly about this time point. Most viewed IV administration as ‘*stronger and quicker treatment*’ [Patient17_SiteA_Female_60 years old] required to respond to more severe illness and so viewed the switch positively as an indicator they may soon be well enough to go home. A minority of patients felt their illness was protracted because they had been switched too soon, ‘*maybe I would have got better a lot quicker if they did keep me on the intravenous antibiotics rather than [switch to] oral*’ [Patient 14_SiteA_Female_30 years old]. Consequently, some patients welcomed more information about the rationale for switching and reassurance on the efficacy of treatment(s).

#### Stopping/changing antibiotics: responding to microbiological investigations

Clinicians were cautious about permitting patients scope to inform antibiotic treatment cessation, ‘*I don’t think I would ever want to give the patient a choice to do something that's against antibiotic guidance*’ [Clinician14_SiteB_Medicine Consultant] and again argued this is a clinically driven decision that precludes patient involvement. Nevertheless, some clinicians noted the importance of addressing the discontinuation of antibiotics, particularly in cases of prolonged use.

Patients likewise welcomed more information at this time point, particularly as an opportunity to address their beliefs about antibiotics and ameliorate their uncertainties, ‘*just the reasons why really just so then you’re* [not] *sat there wondering, thinking of questions and they’re not being answered*’ [Patient14_SiteA_Female_30 years old]. But overall, this was not a salient time point for patients.

#### Discharge/home management

Clinicians felt discharge was most suitable for a discussion as patients will be feeling better marking a critical point where responsibility for patient treatment changes hands, ‘*I think it’s really important to make sure that patients can cope with what you want to, how you want to manage it*’ [Clinician5_SiteA_Medince Registrar]. However, others were concerned the nuance needed to permit meaningful discussion may be too onerous for some patients and suggested being led by patients in the extent of discussions.

Patients particularly welcomed more information about the practicalities of self-management at discharge and one patient argued clinicians should prioritize ‘*explaining the risks if you don’t carry on with the antibiotic*’ [Patient14_SiteA_Female_30 years old] to encourage patient adherence to the self-management of treatment(s) at home.

## Discussion

### Summary

Neither patients nor clinicians had a good understanding of what SDM is, but both groups challenged the amenability of SDM in antibiotic prescribing in the hospital setting. First, clinicians were viewed as the main decision makers and patients did not seek further involvement. Second, the necessity of treating acutely unwell patients quickly precluded patient involvement in decision making. Third, antibiotic decisions were viewed as either straightforward or too complex for shared input. Nevertheless, opportunities for improved communication, specifically bi-directional information provision, were identified using the SSTF framework.

### Comparison with existing literature

While clinicians in this study expressed familiarity with SDM, most offered an incorrect definition; one which emphasized decision making within a multidisciplinary team with little or no input from patients. Similar findings have been reported previously^19,20^ where clinicians demonstrated poor understanding of the benefits of SDM and prioritized efficient teamwork over patient inclusion. Echoing findings from previous studies,^21–23^ clinicians in this study positioned themselves as the main decision makers in antibiotic prescribing, citing the importance of clinical judgement. Only a minority supported greater patient involvement, albeit within limits, noting the need to guide patient towards the ‘right decision’. This contrasts with Italian research where hospital clinicians were more open to follow their patient’s lead, including decisions whether antibiotics were prescribed and, if so, the choice of the specific antibiotic regimen.^24^

Our study also identified a tension in perceptions of antibiotic-related decisions. On one hand, they were perceived as straightforward, supported by clear guidance with binary options thus not needing patient input. On the other hand, they were also described as involving nuanced judgements, which made it difficult to involve patients. Consistent with older^25^ and more recent studies from both secondary^26,27^ and primary care,^28^ patients in this study received little information about, and had no input into, their antibiotic treatment. Unlike previous studies, however, patients in this study accepted this and were unmotivated to engage clinicians about their treatment. This acquiescence probably stemmed from the high degree of trust patients had in clinicians, which others have likewise reported leads to deference to clinicians’ expertise and viewing them as the ultimate decision makers.^26,29,30^

While most patients reported there were opportunities to ask questions about their care with healthcare professionals most did not. There were several reasons for this, namely, they did not have any questions to ask, they did not think they could meaningfully contribute to discussion, or else they felt powerless in the patient–prescriber dynamic to ask questions. Others have likewise pointed out patients need knowledge and power to contribute to SDM, both of which are currently lacking and must be addressed to change behaviour.^31^ Nevertheless, a small subset of patients, particularly those who had experienced adverse effects from antibiotics in the past or had chronic health conditions, were more inclined to initiate discussions about side effects; an observation supported by others.^32^ In addition, patients seldom mentioned AMR suggestive this was not something they were aware of and/or concerned them in this context: findings consistently reported in literature reviews.^33,34^

While patients singled out admission as a critical moment to shape treatment expectations and establish communication around antibiotic use, patients welcomed improved information provision and bi-directional conversation across SSTF timepoints.^30^ Patients expressed a clear desire for more information to ameliorate the vulnerability they felt being in hospital and to dispel misunderstandings or uncertainties regarding their antibiotic treatment. Discharge, on the other hand, was seen as a more practical and timely point for clinicians, who emphasized the importance of treatment adherence at home.

### Clinical implications

Despite being championed by the UK Department of Health and NICE, our findings highlight clinicians have a poor understanding of what SDM is and most were resistant to patients being more involved in antibiotic prescribing decision making. Others have commented that challenging the well-meaning but misguided clinical view of acting in the ‘patient’s best interest’ is a key barrier to attitudinal change required to embed SDM in practice.^35^ Addressing this gap in knowledge (and potentially skills) should therefore be the first priority, with formal training being welcomed by the clinicians in this study. Accumulating evidence suggests that educational interventions in SDM are well received by clinicians and promote SDM^36–38^ signalling a promising avenue for future research in secondary care settings. It is also noted that patients often seem to mirror the view that ‘doctor knows best’ and underestimate the potential value of their contribution or preferences, which often led to not asking questions. Creating the environment for normalizing patient questions, avoiding jargon, and actively inviting preferences may be helpful to counterbalance patients’ worries about the limited value of their questions of contributions. This can be particularly crucial in the initial stages to set the tone for the remaining interactions.

Some clinicians felt antibiotics-related decisions are different from other medical issues due to black-and-white or too-complex decisions. This perception may limit the willingness to implement SDM as clinicians may default to unilateral decision making. Adapting SDM strategies from other conditions could help bridge this gap,^5^ and may include exploring patients’ preferences for information and their desired level of involvement, as well as addressing the clinicians’ perceptions through training and decision support tools.^19^

Looking further into the future, our findings identified differing perspectives for patients and clinicians regarding the optimal time points where SDM could be initiated. These differing perspectives highlight the importance of clinicians’ awareness of patient’s motivation to be involved in their care, which can lead to better satisfaction with their care and alleviate fear.^27^

### Strengths and limitations

Recruitment of participants from three different sites representing different regions enhanced transferability of the findings. However, it is acknowledged that fewer participants were recruited from Site C due to competing priorities at the time. This limited not only the capacity of staff to participate in this study but also their ability to actively engage patient participation, instead they were reliant on passive recruitment (i.e. a Participant Information Sheet was included in discharge paperwork), which perhaps was less effective than speaking with patients directly about the study. While we did not identify any differences based on site it is possible this limits the representativeness of our sample. While other studies explored SDM in more general terms, by using the SSTF framework we elicited more nuanced discussions around each time point allowing us to identify opportunities to enhance patient–prescriber communication and decision making that may otherwise have been obscured.

### Conclusions

This study highlighted the difficulties in implementing SDM antibiotic prescribing in hospital settings. Difficulty stemmed from misunderstanding what SDM is, time pressure, antibiotic decisions being often viewed on one hand as straightforward or too complex and inherent trust in clinical judgement. Despite SDM being an ambition of UK Department of Health, it is not well understood. We therefore need the medical workforce upskilled in SDM as a concept before attempting to optimize practice using SSTF.

## Supplementary Material

dlaf228_Supplementary_Data

## Transparency declarations

S.J. has been involved in developing virtual patient simulations to develop clinicians SDM skills. Keele University continues to develop similar simulations for a range of external organizations: S.J. has not received any financial reward for these simulations. S.J. has produced non-promotional educational materials on shared decision making for Astra Zeneca; they personally did not receive remuneration for this work, but their employer, Keele University, did. J.U. has conducted advisory board work for biosimilars, is a director of a Private Limited, Company who has various NHS clients, and his spouse has Healthcare Consultancy and undertakes work NHS clients. S.H. has consulted or received educational support from Advanz, Baxter, Bowmed, Gilead, Pfizer, Shionogi, Tillots. All other authors: none to declare.

## Supplementary data

Supplemental File available as Supplementary data at *JAC-AMR* Online.
