# Supplementary material for: Patient and clinician views on inpatient antibiotic shared decision-making: a qualitative study
Source: JAC Antimicrob Resist. 2026 Jan 7;8(1):dlaf228. doi: 10.1093/jacamr/dlaf228 (PMC12775835; doi:10.1093/jacamr/dlaf228)
Supplement: dlaf228_Supplementary_Data [file dlaf228_supplementary_data.docx]

**SHared decision-making and Antimicrobial stewaRdship in secondary carE: exploring opportunities along the Start Smart then Focus patient pathway (SHARE)**

**Interview Topics Overview (Health Care Professionals)**

**Briefing:**

1) Thank participant for agreeing to take part.

2) Introduce self.

3) This interview is for the SHARE study. Professionals will have difference experiences of providing care to patients. These differences are important to us, and we value your unique perspective.

4) If at any time during the interview you do not wish to answer a question that’s okay.

5) I would like to digitally record our conversation. The recording will be typed out, but everything you say will be anonymous. Your name and any names you mention, and any places you mention will be taken out, so that if someone read your interview, they would not know who you are or where you work.

6) Your interview will remain confidential, unless it is possible that you or someone else is at risk, but this will be discussed with you first. (As discussed and outlined in the consent form)

7) If, at any stage, you wish to stop the audio recorder, please let me know.

8) Do you have any questions?

**Topics to be explored**

Below is a list of topics to be discussed in this study. The work will remain flexible with respect to participants’ agendas. Therefore, we may add new topics as the interviews progress and data collection continues. However, the key topics of clinicians’ views and experiences of shared decision making overall and in relation to antibiotics, the barriers and facilitators to shared decision making, and opportunities to introduce shared decision making in hospitals, perceived benefits and disadvantages will remain the same.

1. Participants’ views and experiences of shared decision making overall and in relation to antibiotic prescribing
2. Participants’ views and experiences of barriers and facilitators to shared decision making
3. Participants’ views of opportunities to introduce shared decision making
4. Participants’ views and experiences of benefits and disadvantages of shared decision making

**Example questions** (additional questions may be added following the topics above):

**Section A**

1. Can you tell me about your role in managing patients with infections in your hospital?

Prompts: What infections do you commonly manage?

1. Within your role of managing infections, what does shared decision making mean to you?

Prompts: What do you understand by shared decision making? What might be preventing shared decision making?

1. Thinking specifically about shared decision making in relation to antibiotics, what are your experiences of shared decision making with patients when managing suspected bacterial infections?

Prompts: What are your experiences of shared decision making with patients in general?

1. How confident do you feel with shared decision making around antibiotic decision making?

Prompts: If confident, what makes you feel confident? How did you gain this confidence?

If not confident, why not? How do you think your confidence might be improved? What support would be useful?

1. What do you think might be the benefits of shared decision making?

Prompts: How do you think shared decision making might benefit patients/prescribers?

What might shared decision making add to the patient experience? What might shared decision making add to your own experience of caring for patients?

1. What do you think might be the disadvantages of shared decision making?

Prompts: Are there any disadvantages patients may experience as a result of shared decision making? Are there any particular patient groups where it may not be suitable? Are there any disadvantages clinicians may experience?

1. Can you tell me about any shared decision making training that you might have received?

Prompts: If some training, how did it come about? What opportunities are there to have training in shared decision making? How useful are these courses? What could make these courses better?

If no training, are you aware of any training? If training is/was available, would you find it useful to engage with it?

**Section B**

We would now like to think about how shared decision making might be undertaken with patients when making antibiotic prescribing decisions. Shared decision making is a collaborative process that supports a person and their healthcare professional to work together to reach a joint decision about tests or treatments, based on evidence and the person's individual preferences, beliefs, circumstances, and values. Often there is more than one option for decisions like which antibiotic to prescribe or how it should be given to the patient. There is usually very little difference in the clinical outcomes of these options, as they all have risks or benefits. Therefore, a patient could be told about the available options and make a decision together with the prescriber.

1. When you think of the whole patient journey from their arrival at hospital through to their discharge and potential management at home, at which points do you think patient shared decision making might take place?

Prompts: Which points do you think are most amenable to patient shared decision making? Which points do you think are least amenable to patient shared decision making? Why?

We would now like you to take a moment to look at the Antimicrobial Stewardship treatment algorithm.


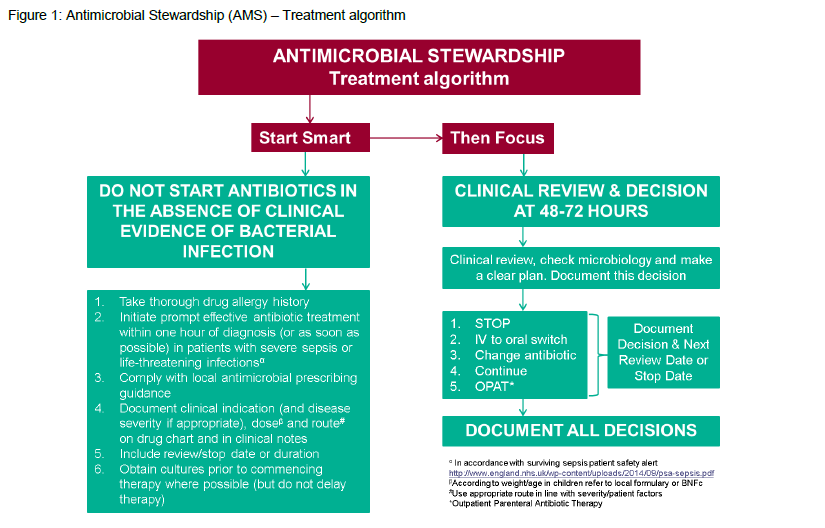


1. [The participant is shown the flowchart] The antimicrobial stewardship treatment algorithm shows various points at which clinical decisions could be made. Which of these points do you think are most amenable to patient shared decision making?

Prompts: Why have you chosen this/these points?

1. Now we are going to consider each of the possible clinical decision points in a bit more detail.

- The first patient shared decision making point is when antibiotics are started.
  - How do you think shared decision making could be included at that stage?
  - How do you think shared decision making could be included in ‘just in case’ prescribing decisions?
  - What challenges might you face with shared decision making at this point?
  - What do you think the benefits of shared decision making at this point might be?
  - What do you think might help to encourage shared decision making at this point? How could your organisation support this way of working?
- The second time point when patient shared decision making might be possible is when antibiotics are switched from being provided by IV to orally.
  - How do you think patients could be included in this prescribing decision?
  - What challenges might you face with shared decision making at this point?
  - What do you think the benefits of shared decision making at this point might be?
  - What do you think might help to encourage and implement shared decision making at this point? How could your organisation support this way of working?
- The third patient shared decision making point might be changing the antibiotic based on diagnostic tests results. This could be a change to a different type of antibiotic, or it could mean stopping antibiotics.
  - How do you think patients could be involved in decisions around early cessation of antibiotic therapy if bacterial infection looks an unlikely cause of the patient’s symptoms?
  - What challenges might you face with shared decision making at this point?
  - What do you think the benefits of shared decision making at this point might be?
  - What support do you think might help to encourage and implement shared decision making at this point? How could your organisation support this way of working?
- Finally, there are several infections that might require intravenous or oral antibiotics for several weeks, e.g. prosthetic joint infection or endocarditis, but these infections can be managed at home by a visiting nurse, if the patient is otherwise well.
  - How do you think patients could be involved in decisions around early discharge and managing their antibiotics at home?
  - What challenges might you face with shared decision making at this point?
  - What do you think the benefits of shared decision making at this point might be?
  - What do you think might help to encourage and implement shared decision making at this point? How could your organisation support this way of working?

1. Overall, which of these prescribing decisions do you think are the most/least amenable to patient shared decision making?

Prompts: Why do think some might be more amenable than others?

1. Which of these prescribing decisions are least amenable to patient shared decision making?

Prompts: why do you think they are not amenable to shared decision making? When might they not be amenable to shared decision making, and why? Is there any way to make them more amenable to shared decision making?

1. Which of the 4 key decision making points (initial prescribing; IV/oral switch; review following diagnostic testing results; discharge/home management) do you think is the most important for patient shared decision making? Which is the least important?

Prompts: Why have you chosen this order?

1. Is there anything else you’d like to add?

- What is your profession (job title) and specialty?
- How many years have you worked as a nurse/doctor/pharmacist/hospital manager?
- How many times a week do you have patient contact?
- Gender

**SHared decision-making and Antimicrobial stewaRdship in secondary carE: exploring opportunities along the Start Smart then Focus patient pathway (SHARE)**

**Interview Topics Overview (Patients)**

**Briefing:**

1) Thank participant for agreeing to take part

2) Introduce self.

3) This interview is for the SHARE study. People will have difference experiences of receiving care in hospitals. These differences are important to us, and we value your unique perspective.

4) If at any time during the interview you do not wish to answer a question that’s okay.

5) I would like to digitally record our conversation. The recording will be typed out, but everything you say will be anonymous. Your name and any names you mention, and any places you mention will be taken out, so that if someone read your interview they would not know who you are or where you work.

6) Your interview will remain confidential, unless (as discussed and outlined in the consent form) it is possible that you or someone else is at risk, but this will be discussed with you first.

7) If, at any stage, you wish to stop the audio recorder, please let me know.

8) Do you have any questions?

**Topics to be explored**

Below is a list of topics to be discussed in this study. The work will remain flexible with respect to participants’ agendas. Therefore, we may add new topics as the interviews progress and data collection continues. However, the key topics of patients’ views and experiences of shared decision making overall and in relation to antibiotics, the barriers and facilitators to shared decision making, and opportunities to introduce shared decision making in hospitals, perceived benefits and disadvantages remain the same.

1. Participants’ views and experiences of recent hospital stay and their involvement in shared decision making
2. Participants’ views and experiences of barriers and facilitators to shared decision making
3. Participants’ views of opportunities to introduce shared decision making
4. Participants’ views and experiences of benefits and disadvantages of shared decision making

**Example questions** (additional questions may be added following the topics above):

**Section A**

**Questions for discussion**

1. I’m interested to hear about your recent hospital stay when you were prescribed antibiotics – are you able to tell me about this?
2. Thinking about your recent hospital stay, can you tell me about any discussions you had with doctors about antibiotics?

Prompts:  Describe/walk through how these were prescribed? What did doctors tell you about antibiotics? Who else did you discuss antibiotics with? Why did you need to take antibiotics? What were you told about how long you might need to be on antibiotics? To what extent were risks and benefits of antibiotics discussed with you? What were you told about your infection? Who did you discuss your infection with? To what extent did you have a chance to ask questions/give input? To what extent do you think you were involved in shared decision making* whilst you were an inpatient?

**if a patient asks what shared decision making is we could say: a joint/collaborative process where a clinician works together with a patient to make a decision about their care*

1. What do you think about being involved in shared decision making about antibiotics?

Prompts: Is this important to you as a patient? How confident would you feel in having those conversations? Do you think you understand the potential benefits and potential risks of each decision? How would you feel about being asked to work with prescribers to make these decisions? What could help you to have these conversations?

1. How do you think shared decision making impacts you as a patient?

Prompts: What might shared decision making add to or take away from your inpatient experience?

1. How do you think shared decision making impacts your doctor? Prompts: How might shared decision making help or hinder prescribers with decision making?

**Section B**

Now we would like to introduce shared decision making when making antibiotic prescribing decisions and would like to talk to you more about this. Shared decision making is a collaborative process that supports a person and their healthcare professional to work together to reach a joint decision about tests or treatments, based on evidence and the person's individual preferences, beliefs, circumstances, and values. Often there is more than one option for decisions like which antibiotic to prescribe or how it should be given to the patient. There is usually very little difference in the clinical outcomes of these options, as they all have risks or benefits. Therefore, a patient could be told about the available options and make a decision together with the prescriber.

There are 4 key antibiotic prescribing moments when shared decision making could take place between prescriber and patient. I would like to explore some of these decisions now in more detail.


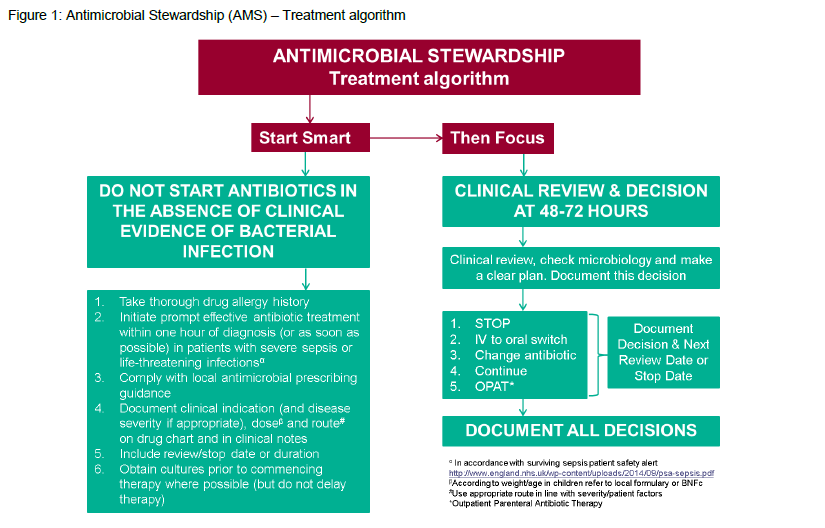


- The first decision point is when antibiotics are started.
  - How important is it for you to be involved in this initial prescribing decision? Have you had any experience of discussing this?
  - What might make shared decision making difficult at this point?
  - What do you think the benefits of shared decision making at this point might be?
  - What support do you think might help encourage shared decision making at this point?

When a patient is admitted to hospital it can be difficult to know if their symptoms are caused by bacteria or a virus. It is important to begin treatment quickly, so antibiotics are often started ‘just in case’ the patient has a serious bacterial infection.

- - Have you had any experience of discussing this? If yes, what were you told about this? Did you have any questions or concerns?
  - How important is it for you to be involved in these ‘just-in-case’ prescribing decisions?
  - What might make shared decision making difficult at this point?
  - What do you think the benefits of shared decision making at this point might be?
  - What support do you think might encourage shared decision making at this point?
- The second time point when shared decision making might be possible is when antibiotics are switched from being provided by IV to orally.
  - Have you had experience of discussing this? If yes, what were you told about this? Did you have any questions or concerns?
  - How important is it for you to be involved in decisions about switching antibiotics?
  - What might make shared decision making difficult at this point?
  - What do you think the benefits of shared decision making at this point might be?
  - What support do you think might encourage shared decision making at this point?
- The third decision point might be changing the antibiotic prescription based on diagnostic tests results. For example, when the clinicians get the test results, they may show that the patient does not need antibiotics. This means that they should be stopped.
  - Have you had experience of discussing this? If yes, what were you told? Did you have any questions or concerns?
  - How important is it for you to be involved in those decisions about stopping antibiotics when a bacterial infection looks unlikely?
  - What might make shared decision making difficult at this point?
  - What do you think the benefits of shared decision making at this point might be?
  - What support do you think might encourage shared decision making at this point?
- Finally, there are several infections that might require intravenous or oral antibiotics for several weeks, e.g., a bone or heart infection. These infections can be managed at home by a visiting nurse, if the patient is otherwise well.
  - Have you had experience of discussing this? If yes, what were you told? Did you have any questions or concerns?
  - How important is it for you to be involved in those decisions about discharge and home management?
  - What might make shared decision making difficult at this point?
  - What do you think the benefits of shared decision making at this point might be?
  - What support do you think might encourage shared decision making at this point?

1. Which of the 4 key decision-making points (initial prescribing; IV/oral switch; review following diagnostic testing results; discharge/home management) do you think is the most important for shared decision making? Which is the least important?

Prompts: Why have you chosen this order?

1. How do you think patients could be encouraged or empowered to start conversations about antibiotic prescribing decision making?

Prompts: What would help you to start this conversion with a prescriber? What would make it difficult to start this conversation?

1. Is there anything else you’d like to talk about that we haven’t covered?

- Age and gender?
- How many inpatient spells in the last 2 years?
- How long was your last stay in hospital?
